# The metaverse digital environments: a scoping review of the challenges, privacy and security issues

**DOI:** 10.3389/fdata.2023.1301812

**Published:** 2023-11-23

**Authors:** Muhammad Tukur, Jens Schneider, Mowafa Househ, Ahmed Haruna Dokoro, Usman Idris Ismail, Muhammad Dawaki, Marco Agus

**Affiliations:** ^1^College of Science and Engineering, Hamad Bin Khalifa University, Doha, Qatar; ^2^Computer Science Department, Gombe State University, Gombe, Nigeria; ^3^Computer Science Department, Gombe State Polytechnic, Bajoga, Nigeria; ^4^Computer Science Department, Federal University of Kashere, Kashere, Gombe, Nigeria

**Keywords:** metaverse, digital environments, security issues and challenges, privacy issues, extended reality, ethical concerns

## Abstract

The concept of the “metaverse” has garnered significant attention recently, positioned as the “next frontier” of the internet. This emerging digital realm carries substantial economic and financial implications for both IT and non-IT industries. However, the integration and evolution of these virtual universes bring forth a multitude of intricate issues and quandaries that demand resolution. Within this research endeavor, our objective was to delve into and appraise the array of challenges, privacy concerns, and security issues that have come to light during the development of metaverse virtual environments in the wake of the COVID-19 pandemic. Through a meticulous review and analysis of literature spanning from January 2020 to December 2022, we have meticulously identified and scrutinized 29 distinct challenges, along with 12 policy, privacy, and security matters intertwined with the metaverse. Among the challenges we unearthed, the foremost were concerns pertaining to the costs associated with hardware and software, implementation complexities, digital disparities, and the ethical and moral quandaries surrounding socio-control, collectively cited by 43%, 40%, and 33% of the surveyed articles, respectively. Turning our focus to policy, privacy, and security issues, the top three concerns that emerged from our investigation encompassed the formulation of metaverse rules and principles, the encroachment of privacy threats within the metaverse, and the looming challenges concerning data management, all mentioned in 43%, 40%, and 33% of the examined literature. In summation, the development of virtual environments within the metaverse is a multifaceted and dynamically evolving domain, offering both opportunities and hurdles for researchers and practitioners alike. It is our aspiration that the insights, challenges, and recommendations articulated in this report will catalyze extensive dialogues among industry stakeholders, governmental bodies, and other interested parties concerning the metaverse's destiny and the world they aim to construct or bequeath to future generations.

## 1 Introduction

The concept of a “metaverse” was first introduced in Stephenson's (1992) science fiction novel “Snow Crash,” in which it is described as a virtual reality shared by millions of users (Stephenson, 2003). The metaverse is a collective virtual shared space, created by the convergence of the internet and virtual reality (VR), where users can interact and engage with each other, as well as virtual objects and environments (Mozumder et al., 2022). It is a virtual world that allows users to create and explore their own digital identities and experiences, and connect with others in real-time. The metaverse is often seen as a potential future evolution of the internet and has been discussed in science fiction literature and media. It is a dynamic and evolving space that offers new opportunities for exploration, creativity, and connection (Allam et al., 2022; Rillig et al., 2022). According to Mystakidis (2022), the Metaverse represents a subsequent realm beyond our current reality, a continual and enduring shared space where physical reality interweaves with digital virtual realms. It results from the fusion of technologies that facilitate immersive engagements with virtual landscapes, digital entities, and individuals, including technologies like virtual reality (VR) and augmented reality (AR).

Recent developments in technology have led to the creation of isolated and fragmented “metaverses” by various institutions and companies. However, most analysts agree that in the long run, these metaverses will evolve toward an integrated and seamless global ecosystem that will affect most human activity across various sectors (Zhang et al., 2022). The recent pandemic and associated restrictions on social behaviors and daily activities have provided an important stimulus for developing novel interaction technologies to reduce the impact of social isolation, and reinforce the idea of an integrated “metaverse” that can substitute standard daily activities even in situations of physical isolation and limited mobility. The metaverse has the potential to be applied in a wide range of sectors, including education (D́ıaz et al., 2020; Locurcio, 2022; Rillig et al., 2022; Suh and Ahn, 2022; Wu and Hung, 2022; Zhang et al., 2022), entertainment and social networking (Bojic, 2022), business (Meepung and Kannikar, 2022), healthcare (Thomason, 2021; Mozumder et al., 2022; Wiederhold, 2022; Yang et al., 2022b), manufacturing (Alpala et al., 2022; Han et al., 2022; Magalhães et al., 2022; Suhail et al., 2022; Yang et al., 2022a), transportation (Njoku et al., 2022; Pamucar et al., 2022), tourism (Allam et al., 2022), military and defense (Baughman, 2022), finance (Bisht et al., 2022; Jung et al., 2022; Katterbauer et al., 2022), real estate (Kun and Zong, 2009; Sulaiman et al., 2020; Nalbant and Uyanik, 2021; Tukur et al., 2022), and tourism (Lee and Kwon, 2017; Napolitano et al., 2017; Kirana, 2021; Allam et al., 2022). There are various techniques and technologies utilized in the development and implementation of the Metaverse (Ning et al., 2021; Mozumder et al., 2022). These include virtual reality (VR) and augmented reality (AR) technologies, which create immersive and interactive virtual environments. Additionally, 3D modeling and animation technologies are used to create detailed and realistic virtual objects and environments. Artificial intelligence (AI) and machine learning technologies are also commonly employed in the Metaverse to create interactive and dynamic virtual experiences. With the recent success of generative adversarial networks (GANs) (Goodfellow et al., 2014), AI also has the potential to generate content (semi-) automatically at scale in the future. Other techniques and technologies include natural language processing and speech recognition, which facilitate more intuitive and user-friendly interactions in the virtual environment. However, creating a metaverse digital environment also poses several issues and challenges. One challenge is to ensure that the virtual environment is immersive and engaging for users (Nalbant and Uyanik, 2021; Tukur et al., 2022). Another challenge is ensuring that the Metaverse is accessible and usable for a diverse range of users, including those with disabilities or those unfamiliar with VR technology (Thomason, 2021; Allam et al., 2022; Mozumder et al., 2022; Yang et al., 2022b; Zhang et al., 2022). Additionally, ensuring compatibility with various systems and hardware is crucial in making the Metaverse accessible to as many users as possible. This requires a high level of technical expertise and resources and can be difficult to ensure system stability and performance across different systems and hardware. This challenge is a result of technical inclusiveness, as different devices and platforms may have varying capabilities and requirements, making it difficult to create a consistent and seamless experience across all of them (Ning et al., 2021; Alpala et al., 2022; Wu and Hung, 2022). Furthermore, standardized representations and protocols for efficient streaming and rendering across different systems and devices are essential for creating a fully immersive and interactive metaverse. This requires the ability to stream and render high-quality 3D graphics and multimedia content in real-time (Tukur et al., 2022). Additionally, there are concerns regarding privacy and security in the Metaverse, as users may inadvertently share sensitive information or personal data (Ning et al., 2021; Abraham et al., 2022; Wang et al., 2022). Ensuring the protection of user data and giving users control over their own information is a crucial consideration in the development of the Metaverse. In this paper, we present and discuss our findings obtained through an extensive scoping review regarding the technical and non-technical challenges, privacy, policy, and security issues of creating the digital environment forming the backbone of the Metaverse, an immersive digital experience that uses both existing and future integrated platforms. We conducted a literature review of research published after the start of the pandemic in early 2020, as the Metaverse received significant attention from academia and industry due to its potential to mitigate the consequences of social restrictions imposed during the pandemic. The research questions addressed in this study include:

- What are the challenges in creating the digital environment of the Metaverse?
- How can the challenges identified within the metaverse digital environment be systematically classified, ranked, and analyzed?
- What policy, privacy, and security issues are raised by the advent of the Metaverse?

The contributions of this study include:

- Identification and analysis of the challenges for the practical implementation of the Metaverse.
- Systematic classification, ranking, and analysis of the collected challenges of the Metaverse digital environments.
- Reflection on the policy, privacy, and security issues arising from the Metaverse.

Prior studies have attempted to survey Metaverse challenges, opportunities, and/or applications with respect to specific industrial sectors (Souza et al., 2021; Allam et al., 2022; Rillig et al., 2022; Sriram, 2022; Suhail et al., 2022), while others focused on identifying relevant security and privacy issues (Abraham et al., 2022; Kwon et al., 2022; Meepung and Kannikar, 2022; Wang et al., 2022). A few studies have compiled and disseminated the technology (and methodology) behind the Metaverse (Ning et al., 2021; Mozumder et al., 2022; Zhang et al., 2022). However, to the best of our knowledge, none of the existing studies have integrated all of the above contributions. Hence, this study can be considered the first comprehensive examination aimed at systematically identifying, classifying, ranking, discussing, and analyzing the challenges and issues within the Metaverse digital environment. Additionally, it delves into the discussion of societal considerations (privacy, policy, security) in relation to various industrial sectors.

The remainder of this paper is organized as follows: Section 2 presents the research methodology we adopted in this study. Section 3 focuses on the definition, classification, ranking, and analysis of the challenges postulated by the literature for the Metaverse. In Section 4, we define and analyze the policy, privacy, and security issues of the Metaverse digital environment collected in this scoping review. We then provide our principal findings, strengths and limitations in Section 6, before concluding (Section 7) this study and presenting future work and our recommendations.

## 2 Methodology

To answer the research questions outlined in the introduction, we follow the procedures outlined in the PRISMA Extension for Scoping Reviews (PRISMA-ScR) (Tricco et al., 2018). These procedures serve as a guide for conducting comprehensive scoping reviews such as this. We carried out our literature search in four major steps:

### 2.1 Search execution

**Figure 1:**
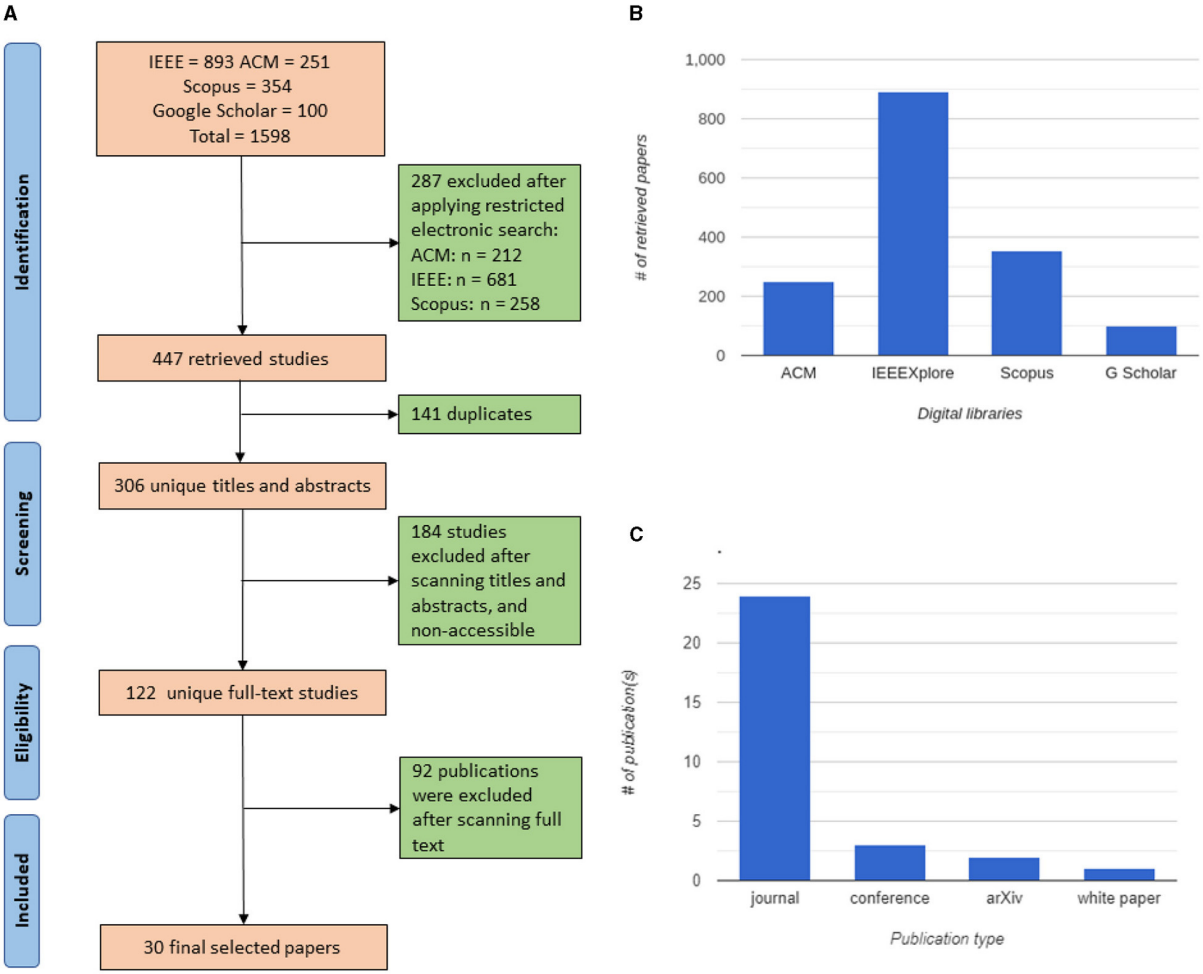
Methodology charts: **(A)** PRISMA chart of the included studies; **(B)** distribution of the initial list of studies per library; **(C)** publication type of the selected papers.

This study uses keyword search queries to identify relevant data sets from the ACM, IEEE, Scopus, and Google Scholar databases. The search procedure, summarized in Figure 1A, is thorough and was carried out in December 2022 using the following keyword search query.

(“virtual environment” OR “digital environment” OR environment OR building OR architecture OR scene) AND (creation) AND (metaverse) AND (challenge* OR issue*)

However, Google Scholar is known to return a large number of results of a wide range of relevance. Therefore, we only consider the first 100 studies listed in the results. Similarly, IEEEXplore returns a large volume of papers. Thus, we initially include publications based on publisher (IEEE) and the most relevant publication venues. This step resulted in 1,598 papers. Figure 1B shows the distribution frequencies and percentages of initial primary studies per library.

### 2.2 Automatic search restrictions and removal of duplicates

In this phase, we include papers published between January 2020 and December 2022 [the pandemic started in December 2019 (WorldMeter, 2022)]. In addition to this time restriction, other filters were applied according to the relevant options available in each digital library. For example, the Scopus library allowed us to filter by date and keywords, while ACM allows to filter by title, date, and publication venue. Table 1 shows the filters and the number of studies retrieved from each digital database after applying library-specific filters. In total, 447 studies were retrieved, and 141 duplicates were detected and removed, leaving 306 unique papers with distinct titles and abstracts.

**Table 1:** Number of papers identified in our restricted electronic search.

**Source**	**Search applied on**	**Retrieved**
IEEEXplore	Initial restrictions: (publisher, and publication title) + date	212
Scopus	Date, and keywords	96
ACM	Title, date, and publication venue	39
Google scholar	Date, and top 100	100
Total		447

### 2.3 Screening based on title, abstract, and full-text availability

The results from the previous phase were further refined based on the titles, abstracts and accessibility of the full papers. This led to the detection and removal of 275 studies with irrelevant titles/abstracts and/or inaccessible full-text, leaving 122 unique full-text studies.

### 2.4 Filtering based on the full-text

We then filtered the results of the previous stages based on their full-text, applying the following inclusion and exclusion criteria. We excluded papers written in a language other than English, conference abstracts, proposals, theses, and dissertations. In addition, articles merely mentioning the term “Metaverse” or its associated techniques instead of actively discussing them (i.e., papers that can rightfully be deemed peripheral to this study) were also removed from our analysis. The included papers were academic research articles from various sources such as journals, conference papers, and white papers from relevant publishers. After carefully reviewing the papers, we retained the thirty (30) most relevant articles as our primary sources. This study is multidisciplinary, and we list the diverse publication venues of the included articles in Table 2. Figure 1C shows the publication types of the included papers. To ensure the authenticity and reduce bias in our findings, three co-authors of this paper carefully conducted the selection. We only kept those publications for our analysis that were independently included by at least two of the co-authors.

**Table 2:** Publication venue of the selected papers.

**#**	**Venue**	**# of publications**
1	ACM Computing Surveys (CSUR)	2
2	Applied Sciences	3
3	arXiv preprint	2
4	British Dental Journal	1
5	Clinical eHealth	1
6	Cyberpsychology, Behavior, and Social Networking	1
7	Electronics	1
8	Environmental Science & Technology	1
9	European Journal of Futures Research	1
10	Extended Reality (XR) and the Erosion of Anonymity and Privacy-White Paper	1
11	Frontiers in Psychology	1
12	Frontiers in Public Health	1
13	Human Centric Computing and Information Sciences	1
14	IEEE Communications Surveys & Tutorials	1
15	International Conference on Advanced Communication Technology (ICACT)	1
16	International Journal of Emerging Technologies in Learning (iJET)	1
17	Journal of Economics, Business and Management	1
18	Journal of Educational Computing Research	1
19	Journal of Intelligence	1
20	Journal of Metaverse	2
21	Military Cyber Affairs	1
22	Nordic Human-Computer Interaction Conference	1
23	Smart Cities	1
24	STAG: Smart Tools and Applications in Graphics	1
25	Technological Forecasting and Social Change	1

## 3 Challenges in creating metaverse virtual environment

There are several challenges that need to be overcome in order to create a metaverse digital environment. In this section, we will present the main definitions, classification, and analysis of the collected challenges from the selected articles.

### 3.1 Collected challenges

The challenges are documented according to the following schema created by us subjectively after analysis of all the reviewed articles:

#### 3.1.1 Hardware and software cost

The establishment of a metaverse digital environment may pose a significant challenge, particularly with regards to the cost of hardware and software. The acquisition of necessary hardware, including servers and computing resources, can entail substantial expenses. Furthermore, the development and maintenance of software for the metaverse, comprising various applications and tools required for user access and interaction, may also entail significant costs. This is especially true for large-scale metaverses with advanced features and a substantial user base (Ning et al., 2021; Allam et al., 2022; Huang et al., 2022; Kwon et al., 2022; Locurcio, 2022; Magalhães et al., 2022; Meepung and Kannikar, 2022; Mozumder et al., 2022; Pamucar et al., 2022; Suhail et al., 2022; Tukur et al., 2022; Yang et al., 2022a; Zhang et al., 2022).

#### 3.1.2 Implementation difficulties

Implementation difficulty is a prevalent challenge in the creation of a metaverse. The project of a metaverse is complex and ambitious, requiring a significant degree of coordination and technical proficiency (McGill, 2021; Ning et al., 2021; Allam et al., 2022; Alpala et al., 2022; Kwon et al., 2022; Magalhães et al., 2022; Suhail et al., 2022; Tukur et al., 2022; Wang et al., 2022; Wu and Hung, 2022; Yang et al., 2022a,b). Among the main challenges of implementing a metaverse are:

- Integration: A significant challenge in the creation of a metaverse is ensuring the seamless interaction of all system components, including the underlying technology that powers the virtual world and the user interfaces and experiences.
- Scalability: The ability to handle a vast number of users and a large amount of data without degradation in performance is crucial for a metaverse, requiring careful planning and the utilization of advanced technologies such as distributed systems and cloud computing.
- Interoperability: The integration of a metaverse with other systems and platforms to allow access from a variety of devices and applications necessitates the development of open standards and APIs to facilitate communication between different systems.
- Time-consuming nature of large-scale implementation: The development and deployment of virtual reality and other technologies required to support a metaverse are complex and costly processes, with the potential for a delay in their widespread availability and adoption. Additionally, the building of a user base and the creation of content for a metaverse can also slow down the process of large-scale implementation.

#### 3.1.3 Digital divides

Digital divides refer to discrepancies in access to and use of digital technology such as the internet and computers across various people and locations. Income, education, geography, and demography may all contribute to these inequalities, resulting in unequal access to the opportunities and advantages of digital technology.

Digital divides can pose a barrier in the setting of a metaverse by preventing some groups of people from accessing and participating in the metaverse. For example, if access to a metaverse involves expensive hardware or software, or a high level of digital literacy, persons who cannot afford or possess the requisite resources or talents may be excluded. This might result in uneven involvement in the metaverse, with certain groups being disproportionately represented or excluded (McGill, 2021; Thomason, 2021; Abraham et al., 2022; Allam et al., 2022; Bojic, 2022; Mozumder et al., 2022; Pamucar et al., 2022; Wang et al., 2022; Yang et al., 2022b; Zhang et al., 2022).

Furthermore, digital inequalities linked with a metaverse might lead to discrimination against people with impairments, particularly those who are blind or have impaired eyesight. For example, if a metaverse's design is not inclusive and does not allow assistive devices such as screen readers, those with visual impairments may be unable to access and participate in the metaverse.

#### 3.1.4 Social control issues

Social control issues can offer a substantial difficulty in the metaverse by making it harder to manage and regulate user behavior in the digital environment (McGill, 2021; Ning et al., 2021; Thomason, 2021; Abraham et al., 2022; Allam et al., 2022; Bojic, 2022; Huang et al., 2022; Pamucar et al., 2022; Suhail et al., 2022; Wang et al., 2022). In a metaverse, some potential social control concerns include:

- Virtual communities: The metaverse may contain a huge number of virtual communities, each with its own set of rules, conventions, and culture, which can make guaranteeing consistency and justice across communities, as well as handling conflicts and disputes, difficult.
- User-generated material: A metaverse may have a large quantity of user-generated content, such as text, photographs, videos, and other media, that is not monitored or controlled by the metaverse's designers. This can make maintaining the quality and appropriateness of this information, as well as enforcing rules and standards for user conduct, difficult.
- Anonymity: Some metaverse users may be anonymous, either by choice or by design, making it impossible to enforce laws and regulations and hold individuals accountable for their conduct.

When creating and executing a metaverse, it is critical to examine these problems since they have the ability to effect the overall user experience and metaverse success.

#### 3.1.5 Ethical and moral issues

The development of a metaverse would raise a number of ethical and moral considerations that must be carefully evaluated (McGill, 2021; Ning et al., 2021; Thomason, 2021; Abraham et al., 2022; Allam et al., 2022; Bisht et al., 2022; Meepung and Kannikar, 2022; Suhail et al., 2022; Wang et al., 2022; Zhang et al., 2022). Some of the key issues that may arise include:

- Dissemination of false information: A metaverse would be a digital place where information could be quickly and readily exchanged and distributed, thereby facilitating the diffusion of false or misleading information, perhaps leading to confusion, disinformation, or injury to people or society. It is critical to verify that information given in a metaverse is correct and trustworthy.
- Integrity issues: A metaverse would be a digital arena where individuals might connect and communicate with one another in new and perhaps unexpected ways, potentially leading to a variety of integrity concerns such as plagiarism, fraud, or other sorts of deceit. To ensure fair and honest interactions, it is critical to create and enforce integrity norms in a metaverse.
- Monopolization of the global market: A successful metaverse might evolve into a global market, with millions of users buying and selling products and services. However, there is a possibility that a few organizations or individuals would achieve a strong position in the metaverse and essentially dominate the market, resulting in unfair competition and perhaps hurting consumers. It is critical to keep the metaverse open and competitive, and to prevent anybody from gaining a dominant position.

On the other hand, Slater et al. (2020) discuss the ethics of realism in virtual and augmented reality (XR) technology. The article emphasizes the need for evidence-based regulation to minimize harm and suggests that developers should consider the risks posed by the content of XR applications, such as games, products for training or therapy, or applications for research. The article also suggests that developers should be transparent about the potential effects of the content on users to limit their legal liabilities and protect individuals from potential harm.

Furthermore, Christopoulos et al. (2021) propose ARLEAN (An Augmented Reality Learning Analytics Ethical Framework) an ethical framework for integrating learning analytics (LA) techniques into augmented reality (AR) applications in education. The ARLEAN framework provides guidelines for developers, instructional designers, and educators on how to integrate LA into their practices to inform their future decisions and support their learners to achieve better results. The article identifies various ethical issues in AR usage that have not been well-documented in the relevant literature and suggests that ethics-informed technological systems can achieve a more widespread adoption.

Moreover, Fernandez and Hui (2022) discuss the ethical and governance issues that arise in the Metaverse, a virtual world where users can interact with each other and digital objects. The article highlights the importance of privacy and data protection in the Metaverse, as well as the need for ethical guidelines and governance structures to ensure the safety and wellbeing of users. The article also suggests that developers and policymakers should work together to create a transparent and accountable framework for the Metaverse that protects users' rights and promotes responsible behavior. In addition, the authors propose a preliminary modular-based framework for an ethical design of the metaverse.

Overall, the ethical and moral implications of a metaverse must be carefully examined and addressed to ensure that the metaverse is a safe and fair environment for all users.

#### 3.1.6 Computational complexity issues

In computer science, computational complexity refers to the amount of resources (time and space) necessary to solve a task. It is a measure of algorithm efficiency and an important factor to consider when creating algorithms and systems. Because of the intricacy and dynamic nature of the world, computational complexity is a huge barrier in a metaverse, necessitating massive computer capacity for real-time modeling and rendering (McGill, 2021; Ning et al., 2021; Alpala et al., 2022; Kwon et al., 2022; Magalhães et al., 2022; Pamucar et al., 2022; Suhail et al., 2022; Tukur et al., 2022; Yang et al., 2022a).

One potential example of computational complexity issue in metaverse is for instance, achieving a high level of detail (LOD) in a metaverse digital environment. The LOD in a metaverse digital environment refers to the amount of detail and complexity that is present in the virtual objects and environments within the metaverse (Kolbe, 2009). Achieving a high level of detail can be challenging for several reasons:

- Computing power: Rendering high-detail objects and environments requires significant computing power, which can be a challenge for some systems.
- Data storage: High-detail objects and environments may require a large amount of data to be stored, which can be a challenge for systems with limited storage capacity.
- Network bandwidth: In a distributed virtual environment, the level of detail may need to be adjusted based on the available network bandwidth to ensure a smooth experience for users.
- Design considerations: Achieving a high level of detail can also be a challenge from a design perspective, as it may require a significant amount of time and resources to create and maintain detailed objects and environments.

Overall, achieving a high level of detail in a metaverse digital environment can be a complex task that requires careful planning and management of technical and design considerations.

#### 3.1.7 Require significant investments in technology, equipment, and research

The development and maintenance of a metaverse requires significant investments in technology, equipment, and research. The creation of a realistic and immersive virtual world necessitates advanced graphics and visual effects, as well as sophisticated algorithms for simulating physics, lighting, and other environmental factors. Furthermore, powerful servers and networking infrastructure are required to support the large number of users potentially accessing the metaverse simultaneously (McGill, 2021; Ning et al., 2021; Bojic, 2022; Huang et al., 2022; Locurcio, 2022; Meepung and Kannikar, 2022; Suhail et al., 2022; Zhang et al., 2022).

#### 3.1.8 Potential solitary physical lifestyles

One possible risk with the usage of a metaverse is that it may lead to more lonely physical lifestyles, with individuals spending less time in real-world interactions with others. This may have a detrimental impact on people's mental and physical health (McGill, 2021; Ning et al., 2021; Thomason, 2021; Allam et al., 2022; Bisht et al., 2022; Pamucar et al., 2022; Wang et al., 2022; Zhang et al., 2022).

Social isolation and detachment from others, for example, can result in emotions of loneliness, which have been connected to bad health consequences such as depression, anxiety, and even an increased risk of mortality. Furthermore, a lack of face-to-face contacts with others might make it more difficult to build and sustain solid social ties, which can give valuable support and resources during challenging times.

Overall, the possible detrimental impacts on mental and physical health produced by a metaverse's solitary physical lifestyle underscore the necessity for thorough research and design considerations to counteract these effects and assure the wellbeing of its users.

On the contrary, while the use of a metaverse may lead to a more solitary physical lifestyle, which can have detrimental effects on mental and physical health, it may also provide benefits for people with disabilities (Allam et al., 2022; Zhang et al., 2022). Here are some additional points to consider:

- Accessibility: The metaverse has the potential to provide a more accessible and inclusive environment for people with disabilities. For example, virtual reality can simulate experiences that may be difficult or impossible for people with physical disabilities to experience in the real world, such as climbing a mountain or exploring a forest. Additionally, the metaverse can provide a more level playing field for people with disabilities to participate in social and economic activities (Allam et al., 2022; Zhang et al., 2022).
- Social interaction: The metaverse can also provide opportunities for social interaction and connection for people who may have difficulty with face-to-face interactions in the real world. For example, people with social anxiety or autism may find it easier to interact with others in a virtual environment where they can control their interactions and environment (Zhang et al., 2022).
- Mental health: The metaverse may also provide benefits for mental health. For example, virtual reality therapy has been shown to be effective in treating anxiety disorders, phobias, and post-traumatic stress disorder. Additionally, the metaverse can provide a sense of community and belonging for people who may feel isolated or disconnected in the real world (Allam et al., 2022).

It is important to consider both the potential risks and benefits of using a metaverse and to design it in a way that promotes wellbeing and inclusivity for all users. This includes addressing issues such as data security, privacy, and informed consent, as well as ensuring that the metaverse is accessible and inclusive for people with disabilities (Thomason, 2021; Zhang et al., 2022).

#### 3.1.9 Intensive energy requirement and negative consequences

One potential challenge associated with the use of a metaverse is the high energy usage that is required to support these virtual environments. The complex technologies involved in a metaverse, such as virtual reality systems and online networks, can be energy-intensive, and their use on a large scale could have negative consequences for the environment (McGill, 2021; Ning et al., 2021; Thomason, 2021; Allam et al., 2022; Rillig et al., 2022; Wang et al., 2022; Yang et al., 2022a). Some potential consequences of the intensive energy requirements of a metaverse include:

- Increased greenhouse gas emissions, which could contribute to climate change.
- Higher energy costs for individuals and businesses using virtual reality technology.
- Strains on the electrical grid and other infrastructure, which could lead to power outages or other disruptions.
- Negative impacts on the environment, such as air and water pollution from the production of electricity.

#### 3.1.10 Potential addiction

Addiction is a possible challenge to the establishment and implementation of a metaverse. A metaverse has the potential to be very engaging and immersive, which may lead to the development of compulsive behaviors in some users (McGill, 2021; Ning et al., 2021; Abraham et al., 2022; Allam et al., 2022; Bojic, 2022; Wang et al., 2022; Zhang et al., 2022).

Addiction to activities such as gaming in a metaverse may have a detrimental impact on a person's productivity and financial wellbeing. Excessive usage of a metaverse can result in low productivity, resulting in reduced income and a higher cost of living. Addicts to metaverse gaming may spend a substantial amount of time and money on the pastime, which might have a severe impact on their financial condition. Addiction to metaverse activities may also result in poorer productivity at work or school, leading in lower income and career possibilities, as well as trouble focusing on tasks and perhaps missing work or school.

#### 3.1.11 Increasing sedentary lifestyle/cyber-syndrome

One possible issue with using a metaverse is that it may contribute to a more sedentary lifestyle (Ning et al., 2021; Thomason, 2021; Abraham et al., 2022; Allam et al., 2022; Pamucar et al., 2022; Wang et al., 2022; Wiederhold, 2022). People may participate in less physical exercise in the real world as they spend more time in virtual worlds. This might result in bad health effects linked with inactivity, such as obesity, heart disease, and other chronic illnesses (Park et al., 2020; Slater et al., 2020; Thomason, 2021).

The term “cyber-syndrome” refers to a physical, social, and psychological illness induced by excessive internet use, which can be aggravated by the high level of involvement in the metaverse (Pamucar et al., 2022). Here are some additional points to consider:

- Digital health: Digital health is revolutionizing healthcare and becoming a critical enabler of change in the pharmaceutical and biotechnology sectors. The COVID-19 pandemic has accelerated the technological mediation of communication on an unprecedented scale, with many physical activities moving to the online space through social media apps, the metaverse, or mobile phones (Thomason, 2021).
- Metaverse: The metaverse is an evolving paradigm of the next-generation internet that aims to build a fully immersive, hyper spatiotemporal, and self-sustaining virtual shared space for humans to play, work, and socialize. Driven by recent advances in emerging technologies such as extended reality, artificial intelligence, and blockchain, the metaverse is stepping from science fiction to an upcoming reality (Wang et al., 2022).
- Sedentary behavior and mental health: Emerging evidence suggests that time spent sedentary may interact with mental health. Sedentary behaviors have been associated with anxiety, depression, and suicidal behavior, and those with sedentary lifestyles were less likely to be physically active. Even with 150 min of weekly exercise, sitting more than 8 hours a day can negatively affect mental health (Hoare et al., 2016).

To mitigate the potential negative effects of using a metaverse, it is important to balance virtual activities with physical exercise and outdoor activities. It is also important to be aware of the signs of cyber-syndrome and take steps to prevent it, such as setting limits on screen time and taking regular breaks from digital devices (Pamucar et al., 2022).

#### 3.1.12 Requires expanding people's knowledge of ICT tools and their management

The necessity for individuals to broaden their understanding of information and communication technology (ICT) technologies and their management is one possible difficulty related with the usage of a metaverse (D́ıaz et al., 2020; McGill, 2021; Meepung and Kannikar, 2022; Suhail et al., 2022; Wu and Hung, 2022; Zhang et al., 2022).

The usage of a metaverse entails a vast range of complicated technologies, and humans will need to constantly broaden their knowledge in order to stay up with the newest discoveries and fully utilize the metaverse. Understanding and using virtual reality technologies, internet networks, and user-generated content are examples of this.

Individuals will also need to have knowledge and skills in the management and administration of these technologies in order to assure optimal metaverse usage and security. They will also need to be able to troubleshoot and address technological issues that may emerge.

#### 3.1.13 Replacing human jobs

The use of a metaverse may lead to the automation of some jobs and tasks, which could have an impact on human employment (McGill, 2021; Allam et al., 2022; Alpala et al., 2022; Bisht et al., 2022; Wang et al., 2022). Here are some additional points to consider:

- Job displacement: The automation of jobs and tasks through the use of a metaverse could lead to job displacement for some workers. However, it is important to note that the impact of automation on employment is not straightforward. While some jobs may be replaced by machines or other automated systems, new jobs may also be created as a result of technological advancements (cnbc.com, 2022).
- Augmentation of human work: The use of a metaverse may also augment the work of human employees rather than fully automating it. For example, AI can be used to assist humans in tasks such as answering questions, writing essays, and arguing legal cases (cnbc.com, 2022). The metaverse can also provide a platform for remote work and collaboration, which can increase productivity and efficiency (hbr.org, 2022).
- New opportunities: The metaverse may also create new opportunities for employment in areas such as virtual reality platforms, gaming, machine learning, and blockchain (hbr.org, 2022). As the metaverse continues to evolve, new roles and skills will be required to support its development and maintenance.

It is important to consider the potential impact of the metaverse on employment and to design it in a way that promotes both technological advancement and human wellbeing. This includes addressing issues such as job displacement, job augmentation, and the creation of new opportunities for employment (Allam et al., 2022; Dwivedi et al., 2022).

#### 3.1.14 Discouragement of experiencing physical environments and neglect of heritage sites

The immersive nature of a metaverse can make virtual environments feel very real, and some individuals may find that they are less interested in exploring and experiencing the real world as a result. This could lead to a reduction in the appreciation and enjoyment of physical experiences, such as the use of the sense of smell, and places, and could potentially have negative impacts on individuals' mental and physical health (McGill, 2021; Allam et al., 2022; Bisht et al., 2022; Rillig et al., 2022; Yang et al., 2022a).

Furthermore, the development of the metaverse also has the potential to lead to the neglect of some heritage sites. As more and more cultural and historical sites are digitized and represented in virtual form, there is a risk that people may become less interested in visiting the physical locations. This could lead to a decline in funding and support for the preservation of these sites, and could ultimately result in their deterioration and loss.

Nevertheless, recent research studies have explored the impact of virtual field trips in the Metaverse or VR on encouraging or increasing intention for visits to physical heritage sites. The following studies provide contrasting eventual impacts:

- Previous studies (Katz and Halpern, 2015; Tussyadiah et al., 2018; Cheng and Tsai, 2019; Rillig et al., 2022) found that virtual reality field trips can increase peoples' interest in visiting physical heritage sites. These studies showed that users who participated in virtual reality field trips were more likely to express interest in visiting the physical sites than those who did not participate (Katz and Halpern, 2015; Tussyadiah et al., 2018).
- Another study published in Smart Cities found that the Metaverse has the potential to enhance urban tourism by creating transformative experiences for visitors to cultural heritage sites (Allam et al., 2022). The study suggests that the Metaverse can make cultural heritage sites more interactive and engaging, which can attract more visitors to the physical sites.
- According to Buhalis and Karatay (2022), mixed reality (MR) can be used to enhance consumer experiences at cultural heritage sites. The study suggests that the Metaverse can be used to create immersive and engaging experiences for visitors to cultural heritage sites, which can increase their interest in visiting the physical sites.
- Another article (Petkov, 2023) suggests that the Metaverse has the potential to revitalize neglected cultural heritage sites by making them more interactive and engaging. The article highlights the importance of governance, ethics, and regulation in managing the Metaverse ecosystem to ensure that it aligns with tourism and cultural heritage preservation.

Overall, the studies suggest that the Metaverse has the potential to enhance the visitor experience at physical heritage sites by creating immersive and engaging experiences. While there is a risk that people may become less interested in visiting the physical sites as more cultural and historical sites are digitized and represented in virtual form, the studies also suggest that virtual reality field trips can increase interest in visiting physical heritage sites. Therefore, it is important to strike a balance between virtual and physical experiences to ensure the preservation and appreciation of physical heritage sites.

#### 3.1.15 Issues related to inappropriate content in the metaverse

The spread of dangerous or inappropriate content in the metaverse is a possible source of worry. As the metaverse grows more prominent, people may come across stuff that they find unsettling or objectionable, such as violent or graphic images, racist or sexist rhetoric, or pornographic stuff. To address this issue, metaverse developers must set clear norms and laws governing the sort of material authorized in virtual spaces, as well as effective means for enforcing these rules and eliminating improper information. It is also critical to educate users on how to report and flag harmful or improper content (McGill, 2021; Abraham et al., 2022; Pamucar et al., 2022; Wang et al., 2022).

#### 3.1.16 Incompatibility issues

Incompatibility issues within the metaverse can occur when virtual environments and platforms are unable to function seamlessly with one another. This can restrict users from accessing and engaging with all the content and experiences available within the metaverse and impede its overall potential. Causes of incompatibility issues include, but are not limited to, variations in technology and software, variations in user interface and design, and variations in the manner in which different platforms handle data and user information (Ning et al., 2021; Alpala et al., 2022; Wu and Hung, 2022).

#### 3.1.17 Connectivity issues

Connectivity challenges are a potential obstacle in the advancement of the metaverse. For the metaverse to operate effectively, users must have access to a stable and high-speed internet connection. However, not all regions have dependable internet access, and even in regions with adequate connectivity, network disruptions and outages can occur. This can impede users from accessing the metaverse and participating in virtual activities (Alpala et al., 2022; Kwon et al., 2022; Wu and Hung, 2022).

#### 3.1.18 Ensuring an immersive and engaging environment for users

Maintaining an immersive and compelling environment for users is a crucial problem in the creation of the metaverse (Meepung and Kannikar, 2022; Tukur et al., 2023), since it demands finding a balance between realism and usefulness. The metaverse must be realistic and credible to users in order to be genuinely immersive (Meepung and Kannikar, 2022). This realism, however, can sometimes jeopardize functioning. A highly complex and realistic virtual environment, for example, may need a substantial amount of computational resources and be difficult for people to traverse and engage with. A simplified and streamlined virtual world, on the other hand, may be more functional but not as immersive and entertaining for users. It is critical for metaverse designers to strike a balance between realism and practicality in order to create virtual places that are both immersive and engaging for users (Nalbant and Uyanik, 2021; Meepung and Kannikar, 2022; Tukur et al., 2022).

Furthermore, in addition to realism, there are other factors that contribute to immersion in virtual environments, such as actional, social, and narrative immersion (Matovu et al., 2023; Mystakidis and Lympouridis, 2023). Actional immersion refers to the feeling of being in control of one's actions within the virtual environment, while social immersion involves feeling connected to other users and the virtual world (Matovu et al., 2023; Mystakidis and Lympouridis, 2023). Narrative immersion is the feeling of being part of a story or plot within the virtual environment (Matovu et al., 2023; Mystakidis and Lympouridis, 2023).

To create an immersive and engaging environment for users, metaverse designers should consider these different types of immersion and strive to balance them with realism and practicality. Some best practices for designing interactive and immersive experiences that engage users include:

- Understanding the audience: Conduct thorough research and user testing to gain insights into users' behaviors and expectations. This understanding will guide the design process, ensuring that the experience resonates with the audience (Furtado, 2023).
- Incorporating meaningful interactivity: Provide users with opportunities to actively engage with the content and influence the outcome. Incorporate interactive elements such as touch, gestures, or voice commands that are intuitive and enhance the overall experience (Tukur et al., 2022; Furtado, 2023).
- Guiding user behavior: Well-designed UI and UX can guide users' actions and help them navigate through the virtual environment effectively. Through visual cues, interactive elements, and clear affordances, designers can direct users' attention and guide them toward specific actions or interactions within the virtual environment (Chowdhari, 2023).
- Reinforcing branding and immersive storytelling: Consistent visual aesthetics, appropriate use of animations and transitions, and the integration of UI elements into the virtual environment can enhance the overall narrative and create a cohesive and memorable user experience (Chowdhari, 2023).
- Ensuring user safety and comfort: Before starting any experience, users should be given clear and simple instructions on how the hardware works, what to expect in the experience, and how to leave an experience if they feel uncomfortable. It is also important to consider users' tech levels and tolerance in terms of duration (Chowdhari, 2023; futurevisual.com, 2023).

By considering these factors and balancing realism with practicality, metaverse designers can create virtual environments that are both immersive and engaging for users.

#### 3.1.19 The requirement for high-standard infrastructure and its maintenance subject to best practices

The necessity for high-standard, adaptive infrastructure to enable enormous data transfer, accept numerous devices and technologies, and be responsive to evolving technology and user behavior are all challenges in metaverse development. It is critical for metaverse success to ensure infrastructure standards (Pamucar et al., 2022; Suhail et al., 2022; Zhang et al., 2022).

#### 3.1.20 Manipulation users' behavior, thoughts and actions

The possible manipulation of users' behavior, beliefs, and actions is a hurdle for the metaverse's progress. As the metaverse grows in popularity, it is feasible that people or groups would exploit it to influence users to their advantage (Ning et al., 2021; Abraham et al., 2022; Meepung and Kannikar, 2022). For example, virtual environments may be designed to encourage specific behaviors or activities, or users could be targeted with customized material designed to influence their ideas and actions. To combat user manipulation in the metaverse, producers of virtual spaces must set explicit norms and laws for the creation and usage of these environments, as well as methods to monitor and enforce these rules. Furthermore, users must be educated on how to spot and protect themselves against possible exploitation and manipulation.

#### 3.1.21 Marginalization of environmental topics

The potential marginalization of environmental topics is a challenge in the advancement of the metaverse. As the metaverse becomes increasingly widespread, there is a possibility that issues related to the environment and sustainability may be relegated to a secondary position in favor of other, more commercially-oriented concerns. This could result in a lack of attention and resources being allocated toward addressing critical environmental issues within the metaverse (Allam et al., 2022; Rillig et al., 2022; Suhail et al., 2022).

#### 3.1.22 Promotion of gentrification

Gentrification may be encouraged by the growth of the metaverse. As virtual spaces grow more popular, specific virtual locations or neighborhoods may become highly sought-after, attracting an overwhelming amount of people and resources. This might result in the displacement of current users and communities, as well as the gentrification of virtual areas. To avoid this consequence, metaverse developers must set norms and laws for the distribution of virtual resources, as well as assure that all users have equitable access to these resources. Furthermore, any possible inequalities or injustices in the allocation of resources inside the metaverse must be monitored and addressed (Allam et al., 2022; Bisht et al., 2022).

#### 3.1.23 Potential risk of urban sprawl

The emergence of the metaverse poses the potential for the replication of urban sprawl. As increasing numbers of individuals begin to engage in virtual activities, there is a risk that virtual cities and neighborhoods may become overpopulated and dispersed, resulting in the inefficient utilization of resources and a decline in virtual inhabitants' quality of life (Allam et al., 2022; Bisht et al., 2022). To mitigate this possibility, it is imperative for metaverse developers to implement strategic planning for virtual cities and neighborhoods, taking into consideration factors such as population density, transportation, and resource allocation. Furthermore, it is essential to continuously monitor the expansion of virtual cities and proactively address any potential issues before they escalate.

#### 3.1.24 The difficulty of achieving a decentralized metaverse

Creating a truly decentralized metaverse is a huge problem. The idea of a decentralized metaverse, with liberty and control for individual users' and groups, is appealing. However, its actual implementation is difficult and time-consuming (McGill, 2021; Thomason, 2021). Coordination of different parties, including technology corporations, users, and governments, is required for the creation of a decentralized metaverse. In addition, extensive technological infrastructure and systems, as well as new governance structures and decision-making processes, must be developed. To summarize, creating a decentralized metaverse is a hard and difficult process.

#### 3.1.25 Measuring presence in virtual environments

Measurement of presence in virtual environments is a significant challenge in the development of the metaverse. Presence, or the feeling of being present in a virtual environment, is a critical aspect of virtual experience immersion and enjoyment. However, the accurate measurement of presence is challenging as it is a subjective and individual experience (McGill, 2021; Souza et al., 2021). To address this challenge, metaverse creators must develop methods and metrics for measuring presence, and incorporate them into the design and evaluation of virtual environments. This could include conducting user studies and surveys, as well as implementing technologies such as virtual reality headsets and haptic feedback systems to enhance presence in virtual environments.

Notwithstanding, there are several methods and metrics for measuring presence, including subjective and objective measures (Souza et al., 2021). Some of the validated frameworks and instruments for measuring presence include:

- The Temple Presence Inventory (TPI): A widely used self-report questionnaire that measures presence in virtual environments (Lombard et al., 2009). The TPI assesses the user's sense of being present in the virtual environment, as well as their level of involvement and emotional response to the experience.
- The Presence Questionnaire (PQ): A self-report questionnaire that measures presence in virtual environments based on four dimensions: spatial presence, involvement, realness, and social richness (Witmer and Singer, 1998). However, Slater et al. (1999) argues that the PQ does not provide a measure of presence that is constructed independently from the factors that might influence it, and that it is a measure of people's responses to various aspects of a system, rather than a measure of presence itself. The author suggests that a measure of presence should be based on a methodology that is directly concerned with the concept of presence, rather than on a questionnaire that measures people's responses to various aspects of a system.
- Neurophysiological measures: These measures use physiological signals such as electroencephalography (EEG) and heart rate variability (HRV) to assess the user's level of presence in virtual environments (Dey et al., 2020).

While these frameworks and instruments provide valuable insights into measuring presence in virtual environments, it is important to note that presence is a subjective and individual experience that can be influenced by various factors such as the user's expectations, prior experience, and the design of the virtual environment (Souza et al., 2021). Therefore, it is crucial for metaverse creators to use a combination of subjective and objective measures, as well as conduct user studies and surveys, to gain a comprehensive understanding of the user's sense of presence in virtual environments (Souza et al., 2021).

In addition to measuring presence, it is also important for metaverse creators to consider the privacy and security implications of collecting user data in virtual environments (McGill, 2021). The IEEE Standards Association has published a report on Extended Reality (XR) Anonymity and Privacy, which provides guidelines for protecting user privacy and security in XR environments (McGill, 2021). By incorporating these guidelines into the design and evaluation of virtual environments, metaverse creators can ensure that users' privacy and security are protected while also providing an immersive and engaging experience.

#### 3.1.26 Skepticism and uncertainty toward metaverse technology

The metaverse is a relatively new and emerging technology, and as with any new technology, there may be concerns about its potential impact on society and individuals. This can lead to skepticism and uncertainty toward its use (Meepung and Kannikar, 2022; Suhail et al., 2022). Potential concerns include:

- Real-world consequences: Some individuals may be concerned about the potential for metaverse challenges to have real-world consequences, such as financial loss or physical harm.
- Cheating: There may be concerns about the potential for cheating in metaverse challenges, either through the use of hacks or other methods of gaining an unfair advantage.
- Fairness and equal access: Some individuals may be concerned that not everyone will have equal access to the technology needed to participate in metaverse challenges, leading to an unequal playing field.

#### 3.1.27 Biased representation of nature

The creation of the metaverse may face difficulties due to biased representations of nature. As the metaverse expands, there is a risk that virtual representations of nature may be prejudiced or incomplete, representing a limited variety of experiences and opinions. This might result in a mistaken perspective of nature, with detrimental effects for the ecosystem as well as human health and wellbeing. It can also result in skewed or distorted depictions of persons, places, and things (Rillig et al., 2022).

#### 3.1.28 Virtual reality fatigue

Virtual reality fatigue is a potential challenge in the use of metaverse technology. Using VR can be mentally and physically demanding, as it involves engaging with a highly immersive and interactive virtual environment. Extended use of VR technology may lead to fatigue, which can manifest in symptoms such as eye strain, headaches, and nausea (Wu and Hung, 2022). To mitigate this, it is important for users to take breaks and rest when using VR technology, and to avoid extended periods of use to prevent fatigue and other negative effects.

#### 3.1.29 Impacts on physical safety and reality awareness

The usage of metaverse technology may pose physical safety and reality awareness issues to users (Abraham et al., 2022). Potential issues include:

- Physical safety: Because the metaverse is immersive and interactive, users may become distracted from their physical surroundings, potentially leading to accidents or injury.
- Reality awareness: Because the metaverse is so deep, users may lose awareness of their real-world surroundings, potentially leading to a separation from reality.

To address these issues, users of metaverse technology must be aware of possible hazards and take actions to reduce them. Setting boundaries and restrictions on the amount of time spent in the metaverse, retaining awareness of the actual world while using technology, and avoiding utilizing technology in potentially harmful situations are all examples of this.

### 3.2 Classification of the metaverse challenges identified by this scoping review

The challenges identified in this scoping review were classified into three categories: Technical, Social/Environmental, and Economical. The classification was based on the most commonly agreed-upon categories, as determined by three co-authors of the paper who independently evaluated the challenges. The categorizations are provided as follows:

#### 3.2.1 Technical challenges

Building the metaverse requires advanced technology and computing infrastructure, including powerful servers, high-speed networks, and sophisticated software tools. This can be a significant challenge, especially for a large-scale metaverse with millions or even billions of users. The technical challenges identified in this review are listed and ranked in Table 5 according to their frequency of occurrences. This includes:

- Implementation difficulties.
- Computational complexity issues.
- Incompatibility issues.
- Connectivity issues.
- Ensuring an immersive and engaging environment for users.
- The difficulty of achieving a decentralized metaverse.
- Measuring presence in virtual environments.

#### 3.2.2 Social and environmental challenges

The metaverse environmental and social challenges include issues such as the energy consumption and carbon footprint of the metaverse, the impact of virtual activities on the real-world environment, and the potential social and psychological effects of extended use of the metaverse. Managing and mitigating these challenges is important for ensuring the sustainability and responsible use of the metaverse. The social and environmental challenges identified in this review are listed and ranked in Table 6 according to their frequency of occurrences. This includes:

- Digital divides.
- Social control issues (difficulties in managing and regulating user behavior).
- Ethical and moral issues.
- Increasing sedentary lifestyle/cyber-syndrome.
- Potential solitary physical lifestyles.
- Intensive energy requirements and negative consequences.
- Discouragement of experiencing physical environments and neglect of heritage sites.
- Issues related to inappropriate content in the metaverse.
- Marginalization of environmental topics.
- Promotion of gentrification.
- Potential risk of urban sprawl.
- Skepticism and uncertainty toward metaverse technology.
- Biased representation of the real world.
- Virtual reality fatigue.
- Impacts on physical safety and reality awareness.

#### 3.2.3 Economic challenges

The economic challenges of the metaverse include issues such as funding the development of the metaverse, generating sufficient revenue to cover costs, creating monetization strategies, ensuring long-term economic sustainability, and competition between various virtual spaces operated by different entities for user and market share. Overcoming these challenges is essential for the success of the metaverse and requires careful planning and management. The economic challenges identified in this review are listed and ranked in Table 4 according to their frequency of occurrences. This includes:

- Hardware and software costs and the risk of a divide in the user base due to varying levels of technological capabilities.
- The need for significant investments in technology, equipment, and research.
- Potential addiction.
- A technological barrier to entry as users need to expand their knowledge of ICT tools and their management.
- Replacing or supplanting existing human jobs.
- The requirement for high-standard infrastructure and its maintenance subject to best practices.

### 3.3 Analysis of the challenges collected in this scoping review

To better understand the potential challenges of creating the metaverse, we used the Constructivist Grounded Theory (CGT) process (Mills et al., 2006) to identify and extract the metaverse challenges discussed in the included studies. This process involves three steps (Charmaz, 2014). First, we performed initial coding, examining the meaning of each challenge and assigning labels to the identified issues. Next, we used focused coding to reduce the number of labeled challenges by creating abstractions of the concepts and identifying their interconnections. Finally, we used theoretical coding to merge similar challenges and consolidate their definitions using our own words to come up with the final challenge categories.

Consequently, following the above procedure, we first identified, named, labeled, and defined 62 challenges, and we computed their occurrences. Then, to reduce any personal bias and to improve the authenticity of the identified issues, two co-authors of this paper cautiously reassessed each challenge. Next, to avoid duplication, similar challenges were merged. Hence, the resulting 62 challenges were reduced to 29 main metaverse challenges.

To further analyze the collected data, we used the recurrence investigation strategy (Niazi et al., 2015; Tukur et al., 2021). This strategy allows us to compute a score for each challenge and report the number of occurrences and percentages of each data variable using a recurrence table. Frequencies are useful for analyzing and comparing data, and can be applied to both ordinal/nominal or numeric information. We tallied the number of times each challenge appeared in the collected papers, and compared the overall significance of each challenge by looking at its occurrence compared to other challenges.

Table 3 lists the identified challenges from the scoping review with their frequencies and percentages. “Hardware and software cost” was cited as a challenge by 43% of our included studies, making it the most common challenge. This is expected considering the high cost of developing and maintaining the necessary hardware and software, and/or the potential barriers to entry for individuals and organizations who may not be able to afford the technology required to participate in the metaverse. This challenge, “Hardware and software cost”, is also identified as the top economic challenge in the metaverse, as indicated in Table 4.

**Table 3:** Ranking of the identified metaverse challenges.

**No**.	**Challenge**	**Freq (*n* = 30)**	**%**	**Rank**
1	Hardware and software cost	13	43%	1
2	Implementation difficulties	12	40%	2
3	Digital divides	10	33%	3
4	Social control issues	10	33%	3
5	Ethical and moral issues	10	33%	3
6	Computational complexity issues	9	30%	4
7	Require significant investments in technology, equipment, and research	8	27%	5
8	Potential solitary physical lifestyles	8	27%	5
9	Intensive energy requirement and negative consequences	7	23%	6
10	Potential addiction	7	23%	6
11	Sedentary lifestyle/Cyber-syndrome	7	23%	6
12	Requires expanding people's knowledge of ICT tools and their management	6	20%	7
13	Replacing human jobs	5	17%	8
14	Discouragement of experiencing physical environments and neglect of heritage sites	5	17%	8
15	Issues related to inappropriate content in the metaverse	4	13%	9
16	Imcompatibility issues	3	10%	10
17	Connectivity issues	3	10%	10
18	Ensuring an immersive and engaging environment for users	3	10%	10
19	The requirement for high-standard infrastructure and its maintenance subject to best practices	3	10%	10
20	Manipulation of users' behavior, thoughts and actions	3	10%	10
21	Marginalization of environmental topics	3	10%	10
22	Promotion of gentrification	2	7%	11
23	Potential risk of urban sprawl	2	7%	11
24	The difficulty of achieving a decentralized metaverse	2	7%	11
25	Measuring presence in virtual environments	2	7%	11
26	Skepticism and uncertainty toward metaverse technology	2	7%	11
27	Biased representation of nature	1	3%	12
28	Virtual reality fatigue	1	3%	12
29	Impacts on physical safety and reality awareness	1	3%	12

**Table 4:** Ranking of the metaverse's economical challenges.

**No**.	**Challenge**	**Freq (*n* = 30)**	**%**	**Rank**
1	Hardware and software cost	13	43%	1
2	The need for significant investments in technology, equipment, and research	8	27%	2
3	Addiction	7	23%	3
4	Requires expanding people's knowledge of ICT tools and their management	6	20%	4
5	Replacing human jobs	5	17%	5
6	The requirement for high-standard infrastructure and its maintenance subject to best practices	3	10%	6

The challenge “Implementation difficulties” is cited as a challenge by 40% of our selected studies, making it the second most common challenge. This is also expected as implementation difficulties are a common challenge in many complex projects, and in particular for building an inclusive system such as the metaverse at scale. Technical challenges include scalability, interoperability, and integration, as well as other legal, societal, and economic challenges involved in creating such a vast and complex virtual environment. Furthermore, as highlighted in Table 5, this challenge “Implementation difficulties” is recognized as the primary technical challenge in the metaverse.

**Table 5:** Ranking of the metaverse's technical challenges.

**No**.	**Challenge**	**Freq (*n* = 30)**	**%**	**Rank**
1	Implementation difficulties	12	40%	1
2	Computational complexity issues	9	30%	2
3	Imcompatibility issues	3	10%	3
4	Connectivity issues	3	10%	3
5	Ensuring an immersive and engaging environment for users	3	10%	3
6	The difficulty of achieving a decentralized metaverse	2	7%	4
7	Measuring presence in virtual environments	2	7%	4

The third most common challenges are: “Digital divides,” “Social control issues,” and “Ethical and moral issues.” These challenges were each cited by 33% of our primary sources. This relatively high frequency can be explained by considering that all of these challenges are related to social impact. This underscores the uncertainty associated with the transformative aspects of new technology, especially if it has the potential for far-reaching impact on our society. Moreover, as evidenced in Table 6, the challenges of “Digital divides,” “Social control issues,” and “Ethical and moral issues” are acknowledged as the foremost social and environmental challenges in the metaverse.

**Table 6:** Ranking of the metaverse's social and environmental challenges.

**No**.	**Challenge**	**Freq (*n* = 30)**	**%**	**Rank**
1	Digital divides	10	33%	1
2	Social control issues	10	33%	1
3	Ethical and moral issues	10	33%	1
4	Potential solitary physical lifestyles	8	27%	2
5	Intensive energy requirement and negative consequences	7	23%	3
6	Sedentary lifestyle/Cyber-syndrome	7	23%	3
7	Discouragement of experiencing physical environments and neglect of heritage sites	5	17%	4
8	Issues related to inappropriate content in the metaverse	4	13%	5
9	Manipulation of users' behavior, thoughts and actions	3	10%	6
10	Marginalization of environmental topics	3	10%	6
11	Promotion of gentrification	2	7%	7
12	Potential risk of urban sprawl	2	7%	7
13	Skepticism and uncertainty toward metaverse technology	2	7%	7
14	Biased representation of nature	1	3%	8
15	Virtual reality fatigue	1	3%	8
16	Impacts on physical safety and reality awareness	1	3%	8

In conclusion, the challenges identified in this scoping review highlight the complexity and multifaceted nature of creating a successful metaverse. The challenges identified are technical, social, environmental, and economic in nature, and range from issues such as technical implementation difficulties, to social and ethical concerns about the impact of the metaverse on society. It is important for developers and stakeholders to consider these challenges and take steps to mitigate them in order to ensure the long-term success and sustainability of the metaverse.

## 4 Policy, privacy, and security issues in the metaverse

The rise of the metaverse raises a variety of policy, privacy, and security concerns. These include concerns with access and usage, privacy, and personal information security.

One key policy challenge is establishing who has the right and under what conditions to access and use the metaverse. There are worries regarding access inequity, especially if the metaverse is only available to those who can afford the technology required to participate. Concerns have also been raised concerning the possible use of the metaverse for criminal activities such as the selling of unlawful items or the spread of copyrighted content.

Another crucial concern in the metaverse is privacy. Participants will provide a great deal of personal information, such as their locations, activities, and contact with others. This raises questions regarding how this information is gathered, kept, and utilized, and if it is done in a way that protects individuals' privacy.

In the metaverse, security is also a big problem. There is a danger of data breaches and other security events due to the enormous volume of personal information provided. There are also concerns about hackers gaining access to and manipulating virtual settings in the metaverse.

Overall, the metaverse's policy, privacy, and security challenges are complicated and will need careful analysis and supervision. Because of their complexities, they necessitate a public debate among metaverse users, policymakers, technologists, and operators. It will be critical for individuals involved in the creation and management of the metaverse to handle these challenges responsibly and effectively if the metaverse is to gain widespread acceptance.

### 4.1 Collected policy, privacy, and security issues

In this section, we present a list, definition, discussion, and examples of metaverse policy, privacy, and security issues found in the literature. The issues are documented according to our schema and mapped accordingly.

#### 4.1.1 Enacting rules and principles for the metaverse

There are a number of challenges in enacting the rules and principles that will govern the metaverse (McGill, 2021; Ning et al., 2021; Thomason, 2021; Abraham et al., 2022; Allam et al., 2022; Bisht et al., 2022; Bojic, 2022; Han et al., 2022; Huang et al., 2022; Pamucar et al., 2022; Wang et al., 2022; Wiederhold, 2022; Zhang et al., 2022). Some of these challenges include:

- Lack of consensus among stakeholders on what the rules and principles should be and how they should be enforced, which could lead to confusion and conflict among users.
- Technology limitations in keeping pace with the rapidly evolving nature of the metaverse.
- Legal challenges in developing rules and principles that are consistent with existing legal frameworks.
- Difficulty in enforcing rules and principles uniformly due to the decentralized and global nature of the metaverse.
- The time required for the formulation of laws on various aspects of the metaverse, which may involve changes to several policies.

Overall, enacting rules and principles for the metaverse requires careful consideration, collaboration, and ongoing adaptation to the changing nature of the technology and the needs of its users.

#### 4.1.2 Privacy threats in the metaverse

Participating in the metaverse may compromise individuals' privacy, including location privacy, habits, and living styles, at various stages of the data service life-cycle, such as acquisition, transmission, processing, governance, and storage (McGill, 2021; Ning et al., 2021; Thomason, 2021; Abraham et al., 2022; Allam et al., 2022; Kwon et al., 2022; Meepung and Kannikar, 2022; Pamucar et al., 2022; Suhail et al., 2022; Wang et al., 2022; Yang et al., 2022b; Zhang et al., 2022).

Examples of privacy threats in the metaverse include:

- Real-world location traceability, which might jeopardize persons' privacy if their real-world position can be traced based on their virtual location and actions.
- Threats to digital footprints, such as the theft of virtual assets or the loss of personal information, as a result of hackers and other bad actors accessing and modifying users' digital footprints.
- Privacy leakage in data transmission occurs when the security of data transmission between users and virtual environments is breached, resulting in the loss of personal information.
- Individuals have less control over how their personal information is gathered, utilized, and shared, which leads to possible privacy violations and a loss of confidence in the metaverse.
- Inadequate privacy policies and practices, when the metaverse's rules and principles do not sufficiently handle privacy issues, resulting in a breach of users' privacy and a loss of trust in the metaverse.

#### 4.1.3 Threats to data management

The data generated or collected by wearable devices and users/avatars in the metaverse may be vulnerable to various threats, including data tampering, fraudulent data injection, low-quality user-generated content, challenges with determining ownership/provenance, and potential intellectual property violations. The main issues related to data management in the metaverse include data breaches, misuse of data, data quality issues, and intellectual property violations etc. (McGill, 2021; Ning et al., 2021; Abraham et al., 2022; Allam et al., 2022; Kwon et al., 2022; Pamucar et al., 2022; Suhail et al., 2022; Wang et al., 2022; Yang et al., 2022b; Zhang et al., 2022).

- Data breaches: Because the metaverse is dependent on the transmission and storage of enormous amounts of data, it is vulnerable to data breaches. This might result in the theft of virtual assets or the loss of personal information.
- Misuse of data: The metaverse may potentially create chances for data misappropriation, such as the selling of personal information or the exploitation of data for harmful reasons.
- Data overload: The metaverse creates massive volumes of data, which can be difficult to manage and analyze properly. This may result in a lack of clarity and knowledge regarding the data and how it is used.
- Data quality: The quality of data in the metaverse may also be an issue, as data may be collected from a variety of sources and vary in quality. As a result, erroneous or misleading inferences may be taken from the data.
- Intellectual property violations: It is possible for people in the metaverse to breach intellectual property rights by utilizing or distributing copyrighted information without authorization. This might cause issues with data management and legal issues.

#### 4.1.4 Threats to authentication/access control in metaverse

The identities of users/avatars in the metaverse can be unlawfully taken and impersonated, and there may be difficulties with authentication across different virtual worlds (Abraham et al., 2022; Kwon et al., 2022; Meepung and Kannikar, 2022; Pamucar et al., 2022; Wang et al., 2022; Zhang et al., 2022). The following are some common authentication threats in the metaverse:

- Impersonation/Identity theft/spoofing: In the metaverse, it is possible for an individual to create an avatar or user account that resembles someone else, in order to gain access to restricted areas or information. This could potentially be used for malicious purposes, such as stealing sensitive information or damaging the reputation of the person being impersonated.
- Cross-platform authentication issues: In the metaverse, users may access virtual worlds from a variety of different platforms and devices, such as computers, smartphones, and virtual reality headsets. This can create challenges for authentication, as different platforms may have different authentication mechanisms, making it difficult to ensure that users are who they claim to be across all platforms.
- Password cracking: In the metaverse, it is possible for an individual to use specialized software to guess or crack a user's password, in order to gain unauthorized access to their account. This could be done through brute-force attacks, dictionary attacks, or other means.
- Avatar authentication issues: In the metaverse, people may create and operate several avatars, each with its own distinct identity and features. This might complicate authentication since it may be difficult to authenticate the identity of a specific avatar, especially if the avatar can alter its look or attributes over time.

#### 4.1.5 Potential to interfere with democratic spaces

The metaverse can also be used to create virtual spaces in which individuals engage in democratic activities, such as voting, campaigns, or participating in public debates (McGill, 2021; Bojic, 2022; Han et al., 2022; Wang et al., 2022). However, there are also potential risks and challenges associated with this. For example, the metaverse could be used to manipulate public opinion or interfere with elections by spreading misinformation or propaganda, especially if transparency with respect to automated (bot/AI) accounts and avatars is missing.

Additionally, there are potential threats to the metaverse network infrastructure. These include:

- DDoS attacks: Distributed denial of service (DDoS) attacks occur when a large number of computers or devices are utilized to flood a network or website with traffic in order to overload it and make it inaccessible to legitimate users. A DDoS assault in the metaverse has the capacity to bring entire virtual worlds down or impair access to specific virtual environments.
- SPOF: A Single Point of Failure (SPOF) is a system component that, if it fails or is taken down, causes the entire system to fail or become unavailable in the metaverse. A SPOF in the metaverse might be used by attackers to interrupt access to the metaverse or bring down entire virtual worlds.
- Sybil attacks: Sybil attacks are types of attacks in which an attacker generates many phony identities in an attempt to obtain disproportionate influence or control over a network or system. A Sybil attack in the metaverse might theoretically be used to alter public opinion or interrupt access to particular virtual settings.
- Network outages: In the metaverse, network outages might occur as a result of technological problems, natural catastrophes, or other factors. This might cause access to the metaverse to be disrupted, posing substantial issues for users and the metaverse as a whole.

#### 4.1.6 Users are not aware of what, when and why their data is being collected in the metaverse, nor the consequences of that collection

Users of the metaverse need to be aware of the data that is being collected, when it is being collected, and the reasons for its collection in order to understand the potential consequences of said collection, and to make informed decisions regarding their willingness to openly or inadvertently disclose data. This lack of awareness can lead to a number of potential problems, including lack of trust, misuse of data, privacy concerns, as well as lack of transparency (Abraham et al., 2022; Meepung and Kannikar, 2022).

- Lack of trust: If users are not aware of what, when, and why their data is being collected in the metaverse, they may not trust the companies or organizations that are collecting the data. This lack of trust can lead to reduced participation or engagement in the metaverse, as users may be hesitant to share personal information or engage in certain activities.
- Misuse of data: If users are not aware of what, when, and why their data is being collected in the metaverse, they may not be able to control how that data is used or shared. This can lead to the misuse of personal data, such as for targeted advertising or other purposes that users may not be comfortable with.
- Privacy concerns: If users are not aware of what, when, and why their data is being collected in the metaverse, they may not be able to understand or control the potential privacy implications of that data collection. This can lead to increased concerns about surveillance, data breaches, or other privacy-related issues.
- Lack of transparency: If users are not aware of what, when, and why their data is being collected in the metaverse, they may not be able to understand the full scope of the data collection process. This can lead to a lack of transparency, as users may not be able to see how their data is being used or shared, or what the potential consequences of that data collection are.

Furthermore, the issue of biometric psychography, as described in Heller (2021) and Christopoulos et al. (2021) is relevant to the lack of awareness of data collection in the metaverse. Biometric psychography is a type of bodily-centered information that can reveal intimate details about users' likes, dislikes, preferences, and interests (Heller, 2021, 2022). The metaverse is an information treasure trove that can collect data from various dimensions, including user movements, eye-tracking, social interactions, and physiological responses (Moates, 2023). The lack of awareness of data collection in the metaverse can lead to the misuse of personal data, such as for targeted advertising or other purposes that users may not be comfortable with Heller (2021). Heller (2021) proposes a framework that integrates privacy and human rights into the development of future immersive tech applications. Therefore, the issue of biometric psychography highlights the importance of user awareness and privacy protection in the metaverse.

#### 4.1.7 Users do not realize the value of their metaverse data and they do not usually read lengthy license agreements presented to them when signing up to a metaverse platform

Many individuals may not fully comprehend the significance of their metaverse data or the consequences of the lengthy license agreements presented to them upon signing up for a metaverse platform. These license agreements can be challenging to read and comprehend, and many individuals may not take the time to fully peruse them (Abraham et al., 2022; Meepung and Kannikar, 2022). As an example, Manchester-based WiFi company Purple included a joke clause in the terms of their free WiFi offer at a festival in 2017, binding individuals to complete 1,000 h of community cleaning services. According to Guardian (2017), 22,000 individuals accepted the license agreement, with only one person claiming the prize offered by Purple for reading the full license agreement. This situation, though not legally enforceable, illustrates the issue at hand. Lengthy terms of use, while necessary from a legal standpoint, can obscure the rights and obligations associated with the utilization of a specific service, potentially resulting in users inadvertently forfeiting important rights or exposing themselves to legal hazards.

#### 4.1.8 Inability to legally mandate standards across metaverse industries

There is currently no legal structure in place to establish standards across the numerous industries and businesses involved in the development of the metaverse, which is a field that is fast expanding. It may be difficult to make sure that the metaverse is open, equitable, and secure for all users in the absence of legal requirements. Additionally, it may result in disparities in the approaches taken by various businesses and sectors to concerns about user rights, security, and data privacy. Without legal guidelines, there is also a chance that the metaverse may fracture, with various businesses and sectors adopting dissimilar technologies and platforms, creating entrance hurdles and reducing the metaverse's ability to provide users new experiences and opportunities (McGill, 2021).

At this juncture, it is important to mention the essential efforts of the IEEE Standards Association toward the ethical use of XR (Mangina, 2021; McGill, 2021). Drawing insights from these documents (Mangina, 2021; McGill, 2021), it is evident that the absence of legal standards may result in significant disparities in how businesses and industries approach critical issues concerning user rights, security, and data privacy within the metaverse.

Without a structured legal framework, businesses and sectors engaged in metaverse development may adopt disparate technologies and platforms, each with its own set of standards and practices. This fragmentation has the potential to create entry barriers, leading to a fractured metaverse where users encounter difficulties in navigating various platforms and experiences. Furthermore, the absence of standardized legal guidelines can hinder the metaverse's ability to provide users with consistent, secure, and innovative experiences, as businesses may prioritize their interests over broader user welfare.

These studies (Mangina, 2021; McGill, 2021), highlight the importance of establishing legal standards in the metaverse, particularly in sectors such as education and privacy. Such standards can help ensure that user rights are protected, data privacy is maintained, and anonymity is respected in virtual environments. By addressing these concerns through legal mandates, the metaverse can evolve as a more inclusive and user-centric space, fostering trust, and providing a level playing field for both users and industry players. Consequently, the absence of legal standards represents a significant challenge to the metaverse's potential to deliver consistent and equitable experiences for all participants in this rapidly expanding virtual realm.

#### 4.1.9 Erosion of anonymity

Users may experience a sense of anonymity due to the use of avatars and virtual identities in the metaverse, allowing them to engage and express themselves without disclosing their real identities. This anonymity might, however, progressively disappear as more information about users and their metaverse activity is gathered. With the use of this information, comprehensive user profiles that are connected to the identities of those individuals can be created. Users may therefore unknowingly no longer enjoy the same level of anonymity and run the danger of being recognized and possibly targeted depending on their metaverse activity (McGill, 2021). To prevent users against the loss of anonymity in the metaverse, companies and platforms must have strong privacy and data protection mechanisms in place to make sure that user data is gathered, processed, and shared in compliance with user preferences and applicable laws and regulations.

#### 4.1.10 The volume of spaces and inhabitants is exponentially growing, making it difficult to govern the metaverse

The metaverse is expanding quickly, and there are more places and people in them than ever before, making it harder and challenging to control and manage these virtual settings. It can be challenging to design and enforce rules and regulations that are efficient and fair for all users given the size and complexity of the metaverse. Additionally, because there are so many various types of spaces and people inhabiting them in the metaverse, it may be difficult to design a governing strategy that works for everyone. It may be required to create adaptable governance frameworks that can take into account the metaverse's dynamic character and the various requirements of its users in order to meet these difficulties. This might entail adopting decentralized and community-based governance strategies as well as creating standards and best practices tailored to particular industries (McGill, 2021).

#### 4.1.11 Crimes associated with XR are more difficult to penalize

Crimes committed in the context of XR, or extended reality, can present unique challenges when it comes to penalization. Because XR environments are virtual, it can be difficult to determine the location and jurisdiction of a crime, making it challenging to identify and prosecute the perpetrators. Additionally, the anonymity and pseudonymity afforded by XR environments can make it difficult to identify and track individuals who have committed crimes. Furthermore, the potential for crimes to cross national borders and involve multiple jurisdictions further exacerbates the issue of penalization (McGill, 2021). To address these challenges, it may be necessary to develop new legal frameworks and international cooperation mechanisms to ensure that crimes committed in XR environments can be effectively penalized.

#### 4.1.12 Missing opting-out methods

Opting out of particular features or services in the metaverse may not always be an easy or straightforward process. In order to improve the user experience, many metaverse platforms include a variety of features and services. However, some of these capabilities may also gather and use personal data in ways that users may not be aware of or may not be comfortable with. Users occasionally might not be aware that certain features or services are available or might not be aware of how to opt out if they choose not to utilize them. Additionally, it may not always be clear or simple to use the opt-out options that are offered, making it difficult for users to exercise their rights and maintain control over their personal data (Abraham et al., 2022). Metaverse platforms must have clear and simple opt-out options for all features and services that gather and use personal data in order to address this issue. By doing this, people may make decisions about their data and privacy that are well-informed and respect their rights.

### 4.2 Analysis of the policy, privacy, and security issues collected in this scoping review

In order to identify the policy, privacy, and security (PPS) issues related to the metaverse, we followed the Constructivist Grounded Theory process (Mills et al., 2006) (see also Section 3.3). Through this procedure, we first identified and defined 42 PPS issues and computed their recurrences by reviewing relevant studies. Two co-authors of this paper then carefully evaluated each issue to minimize any personal bias and enhance the authenticity of the identified issues. To avoid duplication, similar challenges were subsequently consolidated, resulting in 13 main PPS issues.

To further analyze the identified issues, we used the recurrence investigation strategy (see also Section 3.3) to compute a score for each issue and reported frequencies and percentages of each issue in a recurrence (Table 7). As shown in the table, the most common PPS issue identified was “Enacting rules and principles for the metaverse,” which was covered in 13 (43%) of the included studies. This is expected as the creation of the metaverse would require cooperation from various stakeholders, including technology companies, governments, and individual users, making it challenging to reach consensus on the rules and principles that should govern the metaverse. Additionally, these rules and principles would need to be flexible and adaptable as the technology and use of the metaverse evolves over time. The second most common PPS issue was “Privacy threats in the metaverse,” which was reported in 40% of the included studies. This can be attributed to the fact that in the metaverse, users would likely share personal information, such as their identities, locations, and activities, which could make them vulnerable to privacy threats such as identity theft, harassment, or surveillance.

**Table 7:** Ranking of identified PPS issues.

**No**.	**Issue**	**Freq (*n* = 30)**	**%**	**Rank**
1	Enacting rules and principles for the metaverse	13	43%	1
2	Privacy threats in the metaverse	12	40%	2
3	Threats to data management	10	33%	3
4	Threats to authentication/access control in metaverse	6	20%	4
5	Potential to interfere with democratic spaces	4	13%	5
6	Threats to metaverse network	3	10%	6
7	Users are not aware of what, when and why their data is being collected in metaverse, nor the consequences of that collection	2	7%	7
8	Users don't realize the value of their metaverse data and they don't usually read lengthy license agreements presented to them when signing up a metaverse platform	2	7%	7
9	Inability to legally mandate standards across the metaverse industries	1	3%	8
10	Erosion of anonymity	1	3%	8
11	Crimes associated with XR are more difficult to penalize	1	3%	8
12	Missing opting-out methods	1	3%	8

The third most common PPS issue was “Threat to data management,” which was reported in 33% of the included studies. This is related to the second most common issue as in the metaverse, users would likely generate vast amounts of data through their interactions with each other and with virtual objects and environments. Managing this data effectively would require robust data storage and management systems that can handle large volumes of data and process and analyze it in real-time to support the various applications and services offered in the metaverse.

The remaining issues, such as “Threats to authentication/access control in metaverse,” “Potential to interfere with democratic space,” and “Threat to metaverse network” were also reported in a considerable number of studies. However, the less frequent issues such as “Users are not aware of what, when and why their data is being collected in the metaverse, nor the consequences of that collection” and “Users do not realize the value of their metaverse data and they do not usually read lengthy license agreements presented to them when signing up to a metaverse platform” are still important and should be considered in the development and use of the metaverse. It is crucial for stakeholders to address these issues responsibly and effectively and to present terms and conditions in simple and accessible language for non-legal laypersons.

## 5 Securing the metaverse: the role of post-quantum cryptography

### 5.1 Overview for post-quantum cryptography

Post-quantum cryptography (PQC) refers to a new generation of cryptographic algorithms and protocols designed to be secure against attacks by quantum computers (Kumar and Pattnaik, 2020; Kan and Une, 2021). Quantum computers have the potential to break many of the widely used cryptographic schemes in use today, such as RSA (Rivest Shamir Adleman) and ECC (Elliptic Curve Cryptography), due to their ability to efficiently solve certain mathematical problems that underpin these encryption methods (Kan and Une, 2021; Sharma et al., 2023).

The main motivation for developing post-quantum cryptography is to ensure the security of digital communication and data protection in a future where quantum computers become powerful enough to threaten existing encryption standards (Badhwar, 2021; Sharma et al., 2023). Quantum computers, when they become sufficiently advanced, could potentially solve problems like integer factorization and discrete logarithm, which are at the core of RSA and ECC, much faster than classical computers. This would make these encryption methods obsolete (Kan and Une, 2021; Sharma et al., 2023).

Post-quantum cryptography focuses on new mathematical approaches and cryptographic techniques that are believed to be secure against quantum attacks. Some of the key post-quantum cryptographic primitives and algorithms include (Kan and Une, 2021):

- Lattice-based cryptography: uses lattices, regularly spaced point arrays, for public-key cryptography. It relies on hard mathematical problems, such as the Shortest Vector Problem (SVP), and related challenges like Learning with Errors (LWE), making it resistant to quantum computer attacks.
- Code-based cryptography: uses error correcting codes, originally developed for error correction in noisy communication channels, as the basis for public-key encryption. It has been studied for around four decades, with the McEliece algorithm (McEliece, 1978) being a prominent example, and it can be adapted for various security requirements, including IND-CCA through appropriate transformations.
- Multivariate polynomial cryptography: is based on solving multivariate quadratic polynomial equations, known as the MQ problem, with discrete variable values. While finding solutions by computing the Gröbner basis is computationally complex, research has made progress in finding efficient methods, with potential future advancements due to the fundamental significance of the Gröbner basis.

Other categories of Post-Quantum Cryptography (PQC) include isogeny-based cryptography and, or hash-based cryptography, depending upon the foundational mathematical challenges they address (Kan and Une, 2021).

### 5.2 The role of post-quantum cryptography in securing the metaverse

The Metaverse is an extended IoT network that incorporates human beings into the immersive cyber space, allowing more complex interaction. However, with the emerging of Metaverse, the IoT network is extended with new “things”, such as mixed reality equipment, and it is not clear what security and privacy problems could arise in the IoT network extended with Metaverse things (Sensors, 2023). Additionally, quantum computing poses a potential threat to traditional cryptographic methods, making post-quantum cryptography essential for safeguarding metaverse transactions, identity, privacy and confidentiality, trust and verifiability, DeFi platforms, digital property rights, and more. To safeguard the Metaverse's integrity, the adoption of quantum-resistant cryptography becomes imperative (Kwon et al., 2022; Schmidt, 2023). In this context, post-quantum cryptographic solutions are being developed to secure the Metaverse (Kwon et al., 2022). In this article, we explore the role of post-quantum cryptography in securing the metaverse, focusing on six specific topics, each associated with a research paper:

- Curve448 and Ed448 on Cortex-M4 (Anastasova et al., 2022b): The authors explore the implementation of post-quantum cryptographic algorithms on low-power devices such as Cortex-M4. They presents an efficient design for both Curve448 and Ed448 protocols based on Montgomery curve Curve448 and its birationally equivalent Edwards curve. The paper focuses on implementing these protocols on Cortex-M4 microcontrollers, which are low-end IoT platforms. The authors adopted state-of-the-art implementation methods to achieve high performance and low power consumption and also presents a comparison of the proposed design with other existing designs.
- SIKE on Cortex-M4 (Anastasova et al., 2022a): Anastasova et al. present a time-efficient design of finite field arithmetic for the Supersingular Isogeny Key Encapsulation (SIKE) protocol on the Cortex-M4 microcontroller. The authors optimized the finite field arithmetic operations to achieve high performance and low power consumption. They deployed the optimized architecture to the SIKE protocol and observed a latency reduction of 5.63, 3.93, 3.48, and 1.61% for SIKE p434, p503, p610, and p751, respectively, targeting the NIST recommended STM32F407VG discovery board for their experiments. The paper also reports a 5.38% speedup for SIKE p434 compared to the last best-reported assembly implementations. The authors compared their work with other related works and highlighted the importance of secure implementation of post-quantum key exchange schemes.
- SIKE Round 3 on ARM Cortex-M4 (Anastasova et al., 2021): In another contribution, Anastasova et al. present a fast implementation of the SIKE protocol on the ARM Cortex-M4 microcontroller. The authors optimized the implementation of the protocol by using state-of-the-art techniques such as Montgomery multiplication and Karatsuba multiplication. They also used a hybrid approach that combines assembly and C code to achieve high performance and low power consumption. The paper reports a significant improvement in the performance of the SIKE protocol compared to previous works.
- Kyber on 64-Bit ARM Cortex-A (Sanal et al., 2021): Sanal et al. discuss the implementation of Kyber, a post-quantum secure key encapsulation mechanism, on 64-bit ARM Cortex-A processors. The authors aim to provide a compact implementation of Kyber that is suitable for resource-constrained devices such as Internet of Things (IoT) devices. The paper presents a detailed analysis of the performance of the Kyber implementation on ARM64 processors. The authors compare the performance of their implementation with other state-of-the-art implementations of Kyber on different platforms. They show that their implementation achieves a good balance between performance and memory usage, making it suitable for resource-constrained devices.
-     Overall, the paper provides valuable insights into the implementation of post-quantum secure key encapsulation mechanisms on resource-constrained devices. The authors' work on implementing Kyber on ARM64 processors can be useful for researchers and practitioners working on securing IoT devices and other resource-constrained systems.
- Cryptographic accelerators on Ed25519 (Bisheh-Niasar et al., 2021): This paper delves into the development of cryptographic accelerators tailored for digital signatures using Ed25519, a robust elliptic curve digital signature algorithm. The primary objective of the authors is to furnish a high-performance Ed25519 implementation suitable for devices with limited resources, such as those in the Internet of Things (IoT). The paper presents an exhaustive analysis of these cryptographic accelerators' performance, comparing their implementation with other cutting-edge Ed25519 implementations across various platforms. Their results demonstrate that their implementation strikes a favorable balance between performance and memory usage, rendering it apt for resource-constrained devices.
-     In summary, this paper offers valuable insights into the deployment of high-security elliptic curve digital signature algorithms on resource-constrained devices. The work presented on cryptographic accelerators for Ed25519-based digital signatures has the potential to be beneficial to researchers and professionals engaged in fortifying IoT devices and similar systems with limited resources.
- Supersingular isogeny Diffie-Hellman key exchange on 64-bit ARM (Jalali et al., 2017): The paper discusses the implementation of the Supersingular Isogeny Diffie-Hellman (SIDH) key exchange protocol on 64-bit ARM processors. The SIDH protocol is a post-quantum key exchange protocol that is based on the mathematical properties of supersingular elliptic curves and isogenies. The paper presents an optimized implementation of the SIDH protocol on 64-bit ARM processors, which is suitable for resource-constrained devices such as IoT devices. The authors claim that their implementation is faster than the state-of-the-art implementations of the SIDH protocol on other platforms. The paper provides a detailed analysis of the performance of the implementation and compares it with other implementations. The authors conclude that their implementation is efficient and suitable for practical use in resource-constrained devices.

These papers collectively highlight the crucial role of post-quantum cryptography in ensuring the security and privacy of users within the metaverse, where quantum threats are a growing concern. By addressing these specific topics, we shed light on how advanced cryptographic techniques can be leveraged to create a safe and resilient virtual environment for the metaverse's diverse activities and interactions. In conclusion, post-quantum cryptography is an important area of research for securing the Metaverse. Several papers have been published on the implementation of post-quantum cryptographic algorithms on low-power and high-performance devices, which are important for IoT networks and applications that require high-speed cryptography.

### 5.3 The role of NIST lightweight cryptography standardization in securing the metaverse

The National Institute of Standards and Technology (NIST) in the United States has been running a standardization process for lightweight cryptography that can be applicable for post-quantum cryptographic algorithms (Turan et al., 2023). The NIST lightweight cryptography standardization was finalized in February 2023. The aim is to identify and promote secure alternatives to existing cryptographic standards that are vulnerable to quantum attacks (Dobraunig et al., 2021; Turan et al., 2023). While the primary focus of the lightweight cryptography standardization process is not centered on addressing quantum threats, it was still taken into account during the assessment. Generally, the consensus is that most symmetric cryptosystems are deemed to possess a reasonable level of security against potential quantum threats (Turan et al., 2023). The role of NIST's lightweight standardization in post-quantum security for the metaverse can be summarized as follows (Turan et al., 2023):

- NIST lightweight standardization provides a framework for the development of lightweight cryptographic algorithms that can be used to secure low-power devices such as those used in the metaverse.
- The lightweight cryptography standards include algorithms for encryption, authentication, and key establishment that are designed to be efficient and secure, making them ideal for use in the metaverse.
- Post-quantum cryptographic solutions are being developed to secure the metaverse (Kwon et al., 2022; Schmidt, 2023), and NIST standardization is an important step in identifying and promoting secure alternatives to existing cryptographic standards that are vulnerable to quantum attacks.

In addition to the NIST lightweight cryptography standards, there are also lightweight ciphers that include error detection mechanisms that can detect errors in the ciphertext caused by transmission errors or fault attacks. For example, the WAGE stream cipher (Zidarič et al., 2023), the Camellia block cipher (Aoki et al., 2000), the Midori cipher (Banik et al., 2015), and the QARMA block cipher (Avanzi et al., 2023) all include error detection mechanisms.

In conclusion, NIST lightweight standardization can plays a crucial role in post-quantum security for the metaverse. By providing a framework for the development of lightweight cryptographic algorithms that are efficient and secure, NIST standardization can help ensure that the metaverse is protected against quantum threats. However, it is important to note that post-quantum cryptography is an active area of research, and not all proposed post-quantum cryptographic schemes have been widely adopted or standardized. Therefore, it is essential to continue research and development in this area to ensure the long-term security and sustainability of the metaverse.

## 6 Principal findings

In this comprehensive scoping review, we undertake a thorough exploration, classification, ranking, and analysis of the challenges, privacy concerns, and security issues inherent in the development of virtual environments for the metaverse. Our extensive literature review has identified 29 distinct metaverse challenges and 12 pertinent policy, privacy, and security issues. Notably, the top five most prevalent challenges encompass hardware and software costs, implementation complexities, digital disparities, ethical and moral dilemmas, and computational intricacies. These identified challenges have been further categorized into technical, socio-environmental, and economic dimensions. Furthermore, our analysis has revealed that the three most predominant privacy, policy, and security issues include the establishment of governing rules and principles for the metaverse, privacy vulnerabilities, and threats pertaining to data management.

Moreover, in this article, we delve into the implications of post-quantum cryptography (PQC) in bolstering the security of the metaverse, with a specific focus on six key areas: the utilization of Curve448 and Ed448 on Cortex-M4, the implementation of SIKE on Cortex-M4, SIKE Round 3 on ARM Cortex-M4, Kyber on 64-Bit ARM Cortex-A, cryptographic accelerators for Ed25519, and the deployment of Supersingular isogeny Diffie-Hellman key exchange on 64-bit ARM platforms. Additionally, we investigate the role of NIST's lightweight standardization in fortifying the metaverse against quantum threats.

Beyond NIST's lightweight cryptography standards, we also examine lightweight ciphers equipped with error detection mechanisms capable of identifying anomalies in ciphertext resulting from transmission errors or fault attacks. For instance, cryptographic solutions such as Error Detection in the Lightweight Welch-Gong (WG)-Oriented Streamcipher WAGE, fault-resilient architectures in the Camellia block cipher, fault diagnosis procedures for the energy-efficient Midori cipher, and the error detection mechanisms in the block cipher QARMA all contribute to error detection in cryptographic communications.

Overall, this review offers a meticulously documented taxonomy and assessment framework for evaluating the challenges, policy considerations, privacy concerns, and security issues associated with the development of the metaverse and its societal and economic implications. It underscores the imperative nature of addressing these identified challenges and policy, privacy, and security issues in future research and the development of metaverse applications.

### 6.1 Strengths and limitations

This study provides a comprehensive overview of the current state of research on this topic, focusing on the post-pandemic era in which the traditional borders between real and virtual, offline and online spaces have been blurred out of necessity. We believe that human society is more ready than ever to discuss and accept the possibility of a virtual, alternative world such as the metaverse, if said universe can be designed to be a fair, inclusive, and equitable space for its users.

It should be noted, though, that conducting a scoping review is a largely manual process, which means there is a possibility that some relevant studies may have been missed. To reduce this risk, we closely followed the PRISMA guidelines on conducting systematic scoping reviews provided by Tricco et al. (2018). PRISMA extension of scoping reviews utilized in this study is known to be one of the best guidelines for conducting and reporting a high-quality review. Hence, we believe this study is comprehensive and extensive.

However, as for any chosen research method, it is subject to some limitations/validity threats. This research focused on some popular databases including: ACM, Scopus, IEEE and Google Scholar to search and extract the relevant primary studies. It is possible that, other databases might also offer high-quality and relevant studies. However, these libraries were selected due to their reputations and relevance to the research topic.

Furthermore, to ensure the credibility and minimize bias in our findings, multiple co-authors were involved in every step of the process. For example, three co-authors participated in the selection of papers, and only those that were accepted by at least two of them were included in the analysis. Two co-authors also carefully evaluated each challenge to reduce any personal bias and improve the accuracy of our findings. In addition, three co-authors independently classified the challenges, and the final classification was based on the categories that were most commonly agreed upon. This thorough approach was taken to ensure the reliability and validity of our study.

## 7 Conclusion, recommendations, and future work

### 7.1 Conclusion and future work

In conclusion, this scoping review has offered a comprehensive examination of the metaverse digital environments, a domain that has recently captured significant attention as the “next frontier” of the internet. This emerging digital realm holds substantial economic implications for both IT and non-IT industries, yet it presents a complex landscape riddled with intricate challenges, privacy concerns, and security issues.

Throughout our research, we embarked on a meticulous journey spanning the period from January 2020 to December 2022, systematically identifying and analyzing 29 distinct challenges and 12 policy, privacy, and security matters associated with the metaverse. Among the paramount challenges uncovered, concerns related to hardware and software costs, implementation complexities, digital disparities, and ethical and moral dilemmas took center stage. These challenges collectively underscore the multifaceted nature of metaverse development and the hurdles that must be surmounted to navigate this evolving digital frontier.

In the realm of policy, privacy, and security, our investigation revealed the top three concerns: the formulation of rules and principles for the metaverse, the encroachment of privacy threats, and the formidable challenges surrounding data management. These issues demand thoughtful consideration and robust solutions to ensure the ethical and secure evolution of the metaverse.

In summary, the metaverse presents a dynamic and intricate landscape filled with opportunities and challenges. We anticipate that the insights, challenges, and recommendations outlined in this report will serve as a catalyst for extensive dialogues among industry stakeholders, governmental bodies, and other interested parties. These discussions are pivotal in shaping the destiny of the metaverse and the world that we aspire to construct or pass on to future generations. The metaverse is indeed the next frontier, and our collective efforts will determine its course and impact on society and the global economy.

Finally, the NIST lightweight standardization process holds significant potential for enhancing the post-quantum security of the metaverse. Through the establishment of a structured framework for crafting efficient and secure lightweight cryptographic algorithms, NIST's standardization efforts can contribute to fortifying the metaverse against quantum threats. Nevertheless, it is crucial to recognize that post-quantum cryptography remains an evolving research domain, with not all proposed schemes gaining broad acceptance or standardization. Thus, ongoing research and development efforts are imperative to guarantee the enduring security and viability of the metaverse. This highlights the importance of our continued research in this domain for the future of the metaverse.

### 7.2 Recommendations

As concluding remarks, we provide several general recommendations related to the most relevant issues and challenges discussed in this report. Regarding environmental issues, it will be important for the creators of virtual spaces to prioritize sustainability and incorporate environmental concerns into the design and operation of the metaverse. This could include using renewable energy to power virtual environments, designing virtual spaces that encourage sustainable behaviors, and incorporating education and awareness-raising about environmental issues into the metaverse experience. In terms of security issues, the metaverse must be secure to protect the privacy and safety of its users. This requires robust security measures, such as encryption, authentication, and access controls, to prevent unauthorized access and protect against cyber-attacks.

With regards to policy and privacy issues, it is important for the creators of the metaverse to carefully consider and address potential policy and privacy challenges at the planning and development stage. This can include steps such as researching and understanding relevant policies and regulations, engaging with policy makers and stakeholders, developing a clear and transparent policy and privacy framework, and providing ongoing support and guidance to users to help avoid policy violations, and protect their privacy.

Concerning social issues, one recommended approach to address the challenge of digital divides is to ensure that the digital environment is accessible and inclusive for people with disabilities. This can be achieved through the use of accessibility guidelines and standards, such as the Web Content Accessibility Guidelines (WCAG) (Caldwell et al., 2008), and the inclusion of assistive technologies, such as screen readers and text-to-speech softwares.

Finally, to address economic and training issues such as cost and skills required, it may be necessary to take steps such as providing access to affordable hardware and software, offering digital literacy training, and developing inclusive design practices that take into account the needs and abilities of diverse users. This can help to ensure that the metaverse is accessible and inclusive for all users.

## Author contributions

MT: Conceptualization, Data curation, Methodology, Writing—original draft. JS: Conceptualization, Formal analysis, Writing—review & editing. MH: Conceptualization, Formal analysis, Writing—review & editing. AD: Conceptualization, Data curation, Writing—review & editing. UI: Data curation, Methodology, Writing—review & editing. MD: Formal analysis, Methodology, Writing—review & editing. MA: Conceptualization, Formal analysis, Funding acquisition, Project administration, Supervision, Validation, Writing—review & editing.
